# From Purines to Plaques: Serum Uric Acid as a Cardiometabolic Marker and Its Correlation With Dyslipidemia in Essential Hypertension

**DOI:** 10.7759/cureus.92700

**Published:** 2025-09-19

**Authors:** Anbalagan Suyambulingam, Arun K, Abinaya Venkatesan, V Jayakaran, Ebby Perin Mathisha

**Affiliations:** 1 General Medicine, Sree Balaji Medical College and Hospital, Chennai, IND

**Keywords:** cardiovascular disease, dyslipidemia, essential hypertension, hdl, ldl, lipid profile, metabolic risk, serum uric acid, triglycerides

## Abstract

Background

Metabolic disturbances, particularly lipid abnormalities and increased uric acid levels, are commonly observed in essential hypertension and may play a role in endothelial injury and heightened cardiovascular risk. Assessing the relationship between serum uric acid and lipid abnormalities may enhance risk profiling in hypertensive patients.

Methodology

A hospital-based, cross-sectional study was conducted in the Department of General Medicine, Sree Balaji Medical College and Hospital, Chennai, from August 2023 to February 2025. In total, 60 adults aged 35-65 years with essential hypertension (blood pressure >140/90 mmHg) were included. Patients with diabetes, obesity, gout, systemic illnesses, alcohol or tobacco use, or prior lipid-lowering therapy were excluded. After obtaining informed consent, clinical details and anthropometric measures were collected. Fasting samples were analyzed for serum uric acid and lipid profile parameters. Associations between uric acid and lipid fractions were assessed.

Results

Of the 60 hypertensive individuals, 68.3% had hyperuricemia (≥7.0 mg/dL). This group exhibited significantly higher mean triglycerides (195.8 vs. 180.3 mg/dL), low-density lipoprotein (144.4 vs. 132.9 mg/dL), and very-low-density lipoprotein (40.9 vs. 37.1 mg/dL), along with lower high-density lipoprotein (45.2 vs. 47.3 mg/dL) compared to those with lower uric acid levels. Dyslipidemia was more prevalent among hyperuricemic patients (92.6% vs. 78.9%). Serum uric acid also showed a positive correlation with systolic blood pressure and body mass index.

Conclusions

Elevated serum uric acid is significantly associated with adverse lipid profiles in essential hypertension. Hyperuricemia may act as a metabolic marker, underscoring the importance of early detection and combined management of uric acid and lipid abnormalities to reduce cardiovascular risk.

## Introduction

Hypertension, traditionally viewed as a hemodynamic disorder, is now understood to be part of a complex metabolic milieu involving insulin resistance, oxidative stress, and low-grade inflammation [1]. Among the biochemical disturbances associated with hypertension, dyslipidemia, and hyperuricemia, increasing attention has been paid to their potential roles in vascular dysfunction and cardiovascular risk enhancement.

Uric acid, the end product of purine metabolism, has been shown to exert pro-oxidant effects at elevated levels. It interferes with nitric oxide synthesis, promotes vascular smooth muscle cell proliferation, and stimulates the renin-angiotensin-aldosterone system, contributing to endothelial dysfunction and increased vascular tone [2-4]. Hyperuricemia may serve as more than just a metabolic marker; it may be directly implicated in the pathogenesis of essential hypertension.

Simultaneously, dyslipidemia, comprising elevated low-density lipoprotein cholesterol (LDL-C), total cholesterol, and triglycerides, and low high-density lipoprotein cholesterol (HDL-C), is a recognized contributor to atherogenesis and cardiovascular events. These lipid abnormalities are more frequent in individuals with hypertension and may share a mechanistic overlap with hyperuricemia, particularly in insulin-resistant states [5,6].

Recent studies propose that uric acid and lipids are interlinked through several common pathways, including inflammation, adipokine imbalance, renal dysfunction, and altered lipoprotein metabolism [7]. Moreover, obesity and visceral fat accumulation, often seen in hypertensive individuals, may exacerbate both uric acid production and lipid profile derangement.

Despite this theoretical basis, limited data are available from Indian cohorts assessing the direct relationship between serum uric acid and lipid profile components in essential hypertension. Ethnic differences in dietary habits, genetic predispositions, and environmental exposures may modulate this interaction and influence clinical outcomes [8]. Investigating the correlation between serum uric acid levels and lipid profile alterations in a hypertensive population may reveal surrogate markers of vascular risk and offer avenues for early metabolic intervention.

## Materials and methods

Study design and setting

This hospital-based, cross-sectional, observational study was conducted in the Department of General Medicine, Sree Balaji Medical College and Hospital, Chennai. The study duration spanned from August 2023 to February 2025. The study was approved by the Institutional Ethics Committee (approval number: 002/SBMCH/IHEC/2023/1995), and written informed consent was obtained from all participants.

Study population

A total of 60 adult patients diagnosed with essential hypertension were enrolled. All participants were between the ages of 35 and 65 years and had resting blood pressure levels exceeding 140/90 mmHg on at least two separate occasions. Subjects were selected from both inpatient and outpatient departments using a non-random convenience sampling method.

Inclusion criteria

Adults between 35 and 65 years of age with a confirmed diagnosis of essential hypertension were eligible for inclusion in the study. Only patients who were not receiving lipid-lowering therapy at the time of enrollment were considered.

Exclusion criteria

Patients with diabetes mellitus, obesity (body mass index (BMI) ≥30 kg/m²), gout, chronic kidney or liver disease, and systemic illnesses were excluded. Individuals with a history of coronary artery disease, cerebrovascular accident, alcohol or tobacco use, or those taking steroids or lipid-lowering drugs were also not considered for inclusion in the study.

Data collection and anthropometry

A standardized case proforma was used to record demographic data, medical history, lifestyle factors, and anthropometric parameters. Height was measured using a stadiometer, and weight was measured using a calibrated digital scale. BMI was calculated using the following formula: BMI = weight (kg)/height (m)^2^. Blood pressure was measured in a seated position using a calibrated sphygmomanometer, with an appropriate cuff size, after five minutes of rest. Three readings were taken at 30-minute intervals, and the mean value was recorded.

Biochemical analysis

Following a 12-hour overnight fast, venous blood samples were obtained from each participant. The serum was separated by centrifugation and subsequently analyzed using standardized enzymatic colorimetric methods. Serum uric acid was estimated using the uricase-peroxidase (Trinder) method, while total cholesterol was determined by an enzymatic endpoint assay. Triglycerides were measured using the glycerol phosphate oxidase method, and HDL-C was quantified by a precipitation technique with polyethylene glycol/dextran reagent. LDL-C and very-low-density lipoprotein cholesterol (VLDL-C) were calculated using the Friedewald formula [9], expressed as LDL-C = TC - HDL-C - (TG/5) and VLDL-C = TG/5. All biochemical investigations were performed in the hospital’s central laboratory, with strict quality control protocols.

Operational definitions

Hyperuricemia was defined as a serum uric acid concentration of 7.0 mg/dL or higher. Dyslipidemia was categorized according to the National Cholesterol Education Program Adult Treatment Panel III (NCEP ATP III) guidelines, which specify total cholesterol greater than 200 mg/dL, LDL-C above 130 mg/dL, HDL-C less than 40 mg/dL in men and less than 50 mg/dL in women, and triglycerides exceeding 150 mg/dL [10].

Statistical analysis

Data were entered into Microsoft Excel (Microsoft Corp., Redmond, WA, USA) and analyzed using SPSS Statistics for Windows, Version 25.0 (IBM Corp., Armonk, NY, USA). Descriptive statistics were used to summarize clinical and biochemical parameters. Continuous variables were expressed as mean ± standard deviation (SD). Associations between serum uric acid levels and lipid profile parameters were analyzed using independent samples t-tests, chi-square tests, and Pearson correlation coefficients. A p-value <0.05 was considered statistically significant.

## Results

A total of 60 patients diagnosed with essential hypertension were included in the study. The mean age of the participants was 50.4 ± 7.9 years. Males constituted 56.7% (n = 34), and females 43.3% (n = 26). The mean BMI was 26.7 ± 3.5 kg/m². Elevated serum uric acid levels (≥7.0 mg/dL) were observed in 41 out of 60 participants (68.3%). Baseline demographic and clinical characteristics are summarized in Table 1. Table 2 presents the comparison of baseline demographic and clinical characteristics between normouricemic and hyperuricemic groups, showing no statistically significant differences in age, gender distribution, BMI, blood pressure, or duration of hypertension. Table 3 compares the lipid profile of patients with and without hyperuricemia. Hyperuricemic patients had significantly higher total cholesterol, triglycerides, LDL-C, and VLDL, while HDL-C was lower but not statistically significant.

**Table 1:** Baseline demographic and clinical characteristics of the study population (n = 60). Values are expressed as mean ± standard deviation (SD) for continuous variables and n (%) for categorical variables. As this table describes baseline characteristics only, no inferential statistical tests were performed.

Parameter	Mean ± SD/n (%)	Statistical test applied
Age (years)	50.4 ± 7.9	Descriptive only (no test applied)
Gender	Male: 34 (56.7%); Female: 26 (43.3%)	Descriptive only (no test applied)
Body mass index (kg/m²)	26.7 ± 3.5	Descriptive only (no test applied)
Systolic blood pressure (mmHg)	152.3 ± 12.1	Descriptive only (no test applied)
Diastolic blood pressure (mmHg)	92.6 ± 8.7	Descriptive only (no test applied)
Duration of hypertension (years)	6.2 ± 3.1	Descriptive only (no test applied)
Hyperuricemia (uric acid ≥7.0 mg/dL)	41 (68.3%)	Descriptive only (no test applied)
Dyslipidemia (any abnormality)	52 (86.7%)	Descriptive only (no test applied)

**Table 2:** Comparison of baseline characteristics between normouricemic and hyperuricemic groups. Values are presented as mean ± standard deviation (SD) for continuous variables and n (%) for categorical variables. The independent t-test was applied for continuous variables, and the chi-square test was used for categorical variables. A p-value <0.05 was considered statistically significant.

Parameter	Uric acid < 7 mg/dL (n = 19)	Uric acid ≥ 7 mg/dL (n = 41)	Test statistic	Statistical test	P-value
Age (years)	49.6 ± 8.1	50.8 ± 7.8	t = -0.51	Independent t-test	0.612
Gender	Male: 10 (52.6%); Female: 9 (47.4%)	Male: 24 (58.5%); Female: 17 (41.5%)	χ² = 0.15	Chi-square test	0.697
Body mass index (kg/m²)	25.9 ± 3.2	27.1 ± 3.6	t = -1.26	Independent t-test	0.213
Systolic blood pressure (mmHg)	150.1 ± 11.8	153.3 ± 12.2	t = -0.93	Independent t-test	0.355
Diastolic blood pressure (mmHg)	91.7 ± 8.6	93.0 ± 8.8	t = -0.54	Independent t-test	0.591
Duration of hypertension (years)	5.9 ± 3.0	6.3 ± 3.2	t = -0.46	Independent t-test	0.645

**Table 3:** Lipid profile in patients with and without hyperuricemia. *: Statistically significant at p < 0.05. Dyslipidemia cut-offs based on the National Cholesterol Education Program Adult Treatment Panel III criteria [10].

Lipid parameter	Uric acid <7 mg/dL (n = 19)	Uric acid ≥7 mg/dL (n = 41)	Test statistic	Statistical test	P-value
Total cholesterol (mg/dL)	198.1 ± 26.4	211.3 ± 29.7	t = -2.08	Independent t-test	0.043*
Triglycerides (mg/dL)	119.3 ± 31.2	165.8 ± 36.7	t = -2.02	Independent t-test	0.049*
Low -density lipoprotein cholesterol (mg/dL)	99.9 ± 24.5	144.4 ± 27.6	t = -2.16	Independent t-test	0.035*
High-density lipoprotein cholesterol (mg/dL)	47.3 ± 5.2	42.2 ± 5.7	t = 1.51	Independent t-test	0.142
Very-low -density lipoprotein (mg/dL)	37.1 ± 6.2	40.9 ± 7.3	t = -2.01	Independent t-test	0.048*

**Figure 1:**
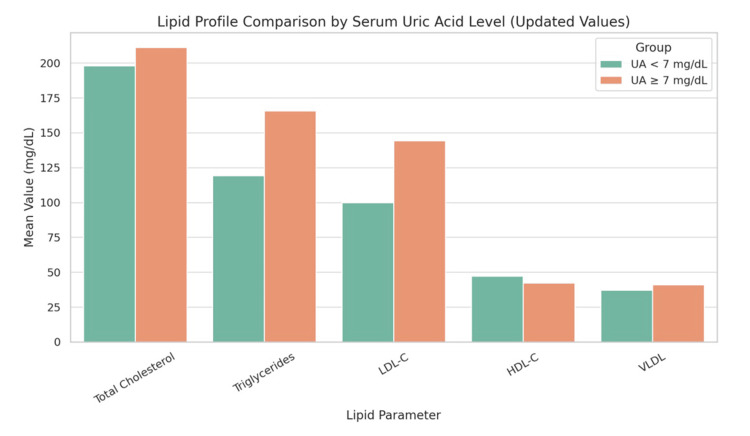
Lipid profile comparison by serum uric acid level. Bar chart comparing mean lipid parameters between primary hypertensive patients with serum uric acid levels <7 mg/dL and ≥7 mg/dL. UA = uric acid; HDL-C = high-density lipoprotein cholesterol; LDL-C = low-density lipoprotein cholesterol; VLDL-C = very-low-density lipoprotein

The distribution of uric acid levels across different subgroups is shown in Figure 1. Patients with elevated uric acid had a significantly higher incidence of hypertension and diabetes compared to the normal uric acid group (p < 0.05).

**Figure 2:**
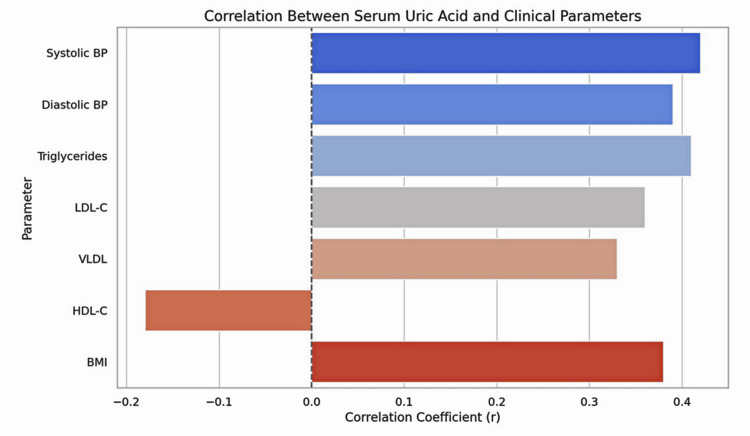
Correlation between serum uric acid and clinical parameters. Correlation coefficients (r values) between serum uric acid and selected clinical/lipid parameters. HDL-C = high-density lipoprotein cholesterol; LDL-C = low-density lipoprotein cholesterol; VLDL-C = very-low-density lipoprotein; BMI = body mass index

**Figure 3:**
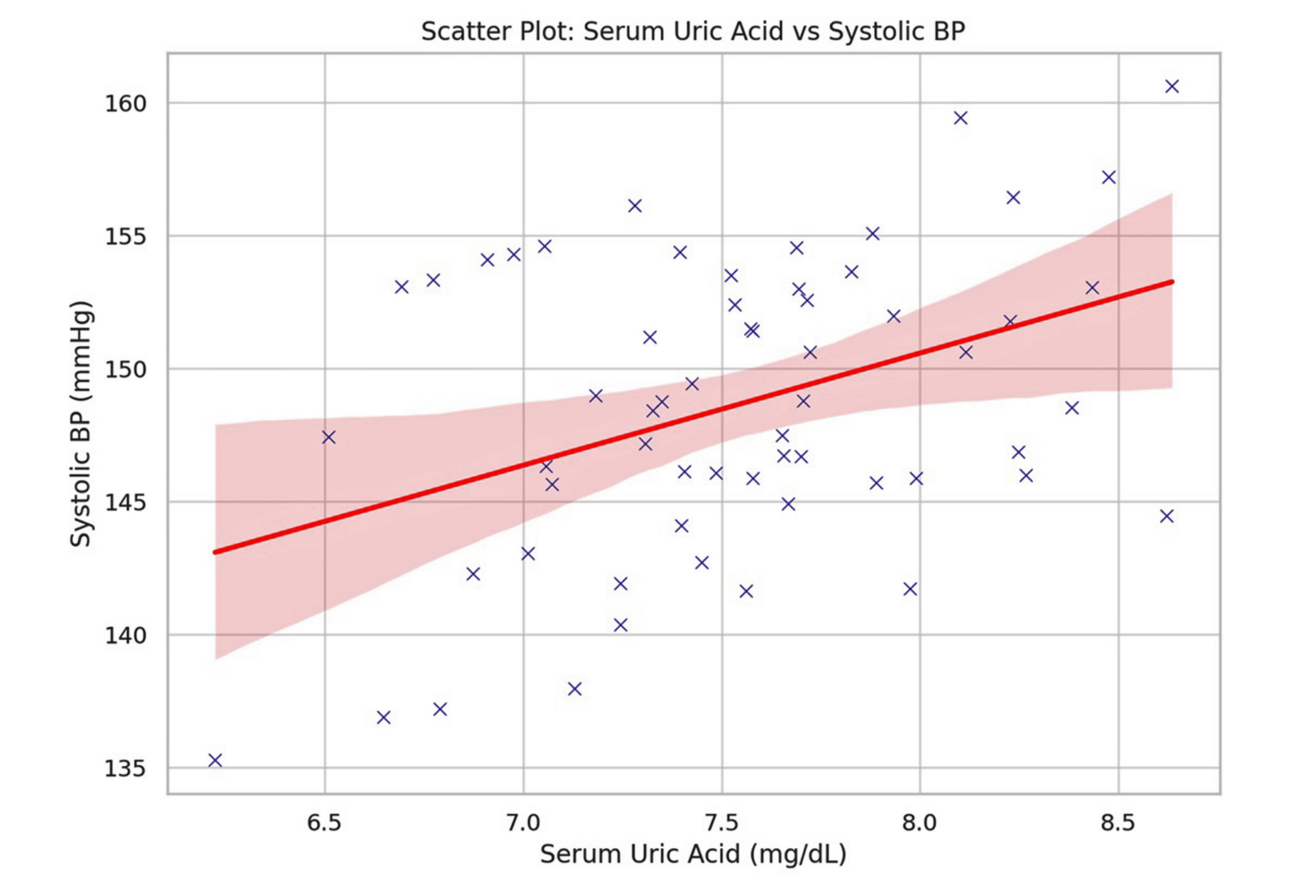
Scatter plot: serum uric acid versus systolic blood pressure. Scatter plot with regression line demonstrating the positive relationship between serum uric acid and systolic blood pressure (simulated data based on observed correlation coefficient r = 0.42).

A correlation analysis (Table 4) showed that serum uric acid positively correlated with systolic blood pressure, diastolic blood pressure, triglycerides, LDL-C, VLDL, and BMI. No significant correlation was found with HDL-C. Figure 2 displays correlation coefficients (r values) between serum uric acid and selected clinical/lipid parameters. Figure 3 shows a scatter plot with a regression line depicting the positive correlation between serum uric acid and systolic blood pressure (r = 0.42, p = 0.002).

**Table 4:** Correlation between serum uric acid and other parameters. *: Statistically significant at p < 0.05. LDL and VLDL were calculated using the Friedewald formula [9].

Parameter	Correlation coefficient (r)	Test statistic	Statistical test	P-value
Systolic blood pressure	+0.42	r = 0.42	Pearson correlation	0.002*
Diastolic blood pressure	+0.39	r = 0.39	Pearson correlation	0.005*
Triglycerides	+0.41	r = 0.41	Pearson correlation	0.003*
Low-density lipoprotein cholesterol	+0.36	r = 0.36	Pearson correlation	0.008*
Very-low-density lipoprotein	+0.33	r = 0.33	Pearson correlation	0.011*
High-density lipoprotein cholesterol	–0.18	r = -0.18	Pearson correlation	0.162
Body mass index	+0.38	r = 0.38	Pearson correlation	0.006*

In this study, 68.3% of patients with essential hypertension were found to have hyperuricemia. These patients exhibited higher mean values of total cholesterol, triglycerides, LDL, and VLDL compared with normouricemic individuals. Although HDL levels were lower in the hyperuricemia group, the reduction was not statistically significant.

## Discussion

This study evaluated the association between serum uric acid levels and lipid profile abnormalities in patients with essential hypertension. Our findings demonstrate that elevated serum uric acid (≥7.0 mg/dL) is significantly associated with higher levels of total cholesterol, triglycerides, LDL-C, and VLDL-C, while HDL-C levels were marginally lower. Furthermore, serum uric acid levels positively correlated with both systolic and diastolic blood pressures, BMI, and lipid fractions, supporting its role as a potential metabolic risk marker.

The prevalence of hyperuricemia in our cohort was 68.3%, which aligns with earlier studies reporting elevated uric acid in 50-70% of hypertensive individuals [1,2]. Elevated serum uric acid has long been suspected to play a role in the development and maintenance of hypertension. It has been shown to induce endothelial dysfunction by inhibiting nitric oxide synthesis and promoting oxidative stress and inflammatory responses in the vascular endothelium [3]. This may increase systemic vascular resistance and contribute to persistent hypertension.

Our study found that patients with hyperuricemia had significantly higher mean triglyceride and LDL-C levels compared to normouricemic individuals, consistent with previous research. For instance, Kuwabara et al. reported a strong correlation between uric acid and triglyceride levels in hypertensive Japanese adults [4]. Similarly, Cicero et al. observed that hyperuricemia was linked to atherogenic lipid profiles and increased cardiovascular risk [5].

The positive correlation observed between uric acid and BMI in our study may reflect the role of adiposity in uric acid production and clearance. Adipose tissue promotes both lipogenesis and uric acid synthesis, while simultaneously impairing renal urate excretion [6]. This is of clinical relevance in South Asian populations, where central obesity often precedes overt metabolic syndrome [7].

Though HDL-C levels were lower in the hyperuricemic group, the difference did not reach statistical significance. However, inverse relationships between HDL and uric acid have been reported in larger population studies [8]. The complex interaction between these parameters is believed to involve oxidative damage to HDL particles and alterations in lipid transport mechanisms in states of hyperuricemia.

LDL-C and VLDL-C values were derived using the Friedewald equation [9], which remains the most widely accepted method in clinical and research settings when triglyceride levels are not excessively elevated. Dyslipidemia was defined according to the NCEP ATP III criteria [10], ensuring standardized thresholds for lipid abnormalities. This methodological consistency strengthens the reliability and comparability of our findings with other studies.

Importantly, our study reinforces the notion that serum uric acid is not merely a by-product of purine metabolism but may be involved in lipid regulation, vascular function, and metabolic stress pathways. Several studies have suggested that uric acid acts as a pro-inflammatory agent in the arterial wall, increasing monocyte chemoattractant protein-1 and C-reactive protein levels [11]. These effects may amplify lipid deposition and vascular remodeling in hypertensive individuals.

From a clinical standpoint, the identification of uric acid as a correlating marker of dyslipidemia may offer additional insights into cardiovascular risk stratification. It also raises the question of whether serum uric acid should be routinely assessed in newly diagnosed hypertensive patients, especially those with features of metabolic syndrome. Further, interventional studies have shown modest reductions in blood pressure with uric acid-lowering agents such as allopurinol in early or mild hypertension, suggesting a potential therapeutic role [12].

A meta-analysis by Grayson et al. confirmed that hyperuricemia significantly increases the likelihood of developing hypertension across multiple populations [13]. Borghi et al. extended these findings by demonstrating that serum uric acid independently predicts cardiovascular and renal outcomes, highlighting its prognostic value in long-term disease progression [14]. In addition, a large Japanese cohort study by Kuwabara et al. showed that individuals with prehypertension and elevated uric acid were more likely to progress to overt hypertension, and this risk was compounded in the presence of metabolic abnormalities such as dyslipidemia [15].

However, it is important to recognize the limitations of our study. The cross-sectional design restricts causal inference. The sample size, though adequate for correlation analysis, limits generalizability. Additionally, we excluded diabetics and obese patients to avoid confounding, which may have narrowed the spectrum of metabolic diversity in our cohort. Longitudinal studies with larger populations are needed to confirm these findings and evaluate the prognostic value of uric acid in predicting cardiovascular outcomes.

## Conclusions

This study highlights a significant association between elevated serum uric acid levels and adverse lipid profile abnormalities in patients with essential hypertension. Hyperuricemia was correlated with higher levels of total cholesterol, triglycerides, LDL-C, and VLDL-C, along with increased systolic and diastolic blood pressures and BMI. These findings reinforce the concept that serum uric acid is not merely a bystander but may act as a metabolic contributor to cardiovascular risk in hypertensive individuals. Given the simplicity and cost-effectiveness of serum uric acid testing, its inclusion in the routine evaluation of hypertensive patients, particularly those with features of metabolic syndrome, may facilitate early risk stratification and more comprehensive management. Future longitudinal and interventional studies are warranted to explore the therapeutic potential of targeting uric acid in the prevention of cardiovascular complications.
